# Prof. Geo. Hadley

**Published:** 1877-12

**Authors:** 


### A Tribute to the Memory of the Late Prof. Hadley.

Prof. Thomas F. Rochester delivered the opening lecture in the course of the Buffalo Medical College on the evening of November 7th, 1877, and at the close of his lecture proper rendered the following

TRIBUTE TO THE MEMORY OF PROF. GEORGE HADLEY.

Gentlemen and Colleagues of the Medical Department of the University of Buffalo: We meet this evening under peculiar circumstances of sadness and solemnity. Since the last re-union of this kind one of the most cherished of our number has passed away from earth forever. One of the original Faculty and founders of this institution, and one who has done much to promote its reputation and its growth by his zeal, his integrity and his remarkable professional attainments. How great indeed is the void produced by the death of George Hadley. How great our loss and how profound our grief—words fail to convey an adequate idea.

To you, sir (Prof. Jame P. White), who alone remain of the original number of the then young men who with equal courage and enthusiasm resolved to establish and to maintain a Medical College in Buffalo (and to resolve was to accomplish), we extend our condolence, on the severance of the last link which united you with a circle of professional brothers, every one of whom was successful, and for the majority of whom we proudly claim eminence and world-wide renown. That this expression is not extravagant it needs but to mention our Faculty of thirty years gone by, naming them in their order on the College records. The Hadleys, James and George, father and son, Charles B. Coventry, James Webster, Charles F. Lee, Frank H. Hamilton, James P. White and Austin Flint. Of this number only the last three are now living—and what their position in the scientific and medical world it needs not my voice to tell. May you, sir, the sole connecting bond remaining to us, between the past and the present association of teachers, long be spared, to guide us by your counsels, to profit us by your teachings, and to be associated with us in this medical school, which you have done more than any other person, past or present, to found, build and sustain.

Peaceful be thy rest, oh friend and brother departed, as is thy memory fragrant and grateful.

Student—No longer from this platform will you hear the gentle voice which has so often spoken to you in truthful and persuasive tones of that science in which it was his delight to give instruction. Never again will you witness that genial smile with which it was his wont to encourage the timid but earnest and studious pupil, or which illumed his contenance on which the beams of quiet wit and humor often played. Those of you who are not now present for the first time know what you have lost, although for the past two years his physical forces were much impaired by the cardiac affection under which he labored, and which, but for his will and his energy would have long since compelled his retirement from public duty. But like every true savant and philanthropist, he courageously persisted in his work and was one of those commended "as faithful to the end." To those of you who only knew Prof. Hadley by reputation, and to no one present was he a stranger in that way, a sketch of his life, necessarily a very brief one, will, I think, be acceptable.

George Hadley was the eldest son of Prof. James Hadley. He was born in Steuben, Oneida county, N. Y., June 20th, 1813. His father was teaching chemistry in the Medical School at Fairfield, Herkimer county, N. Y., and from his very infancy he was as it were surrounded by influences which determined his future career. His father's labratory was his early school and playground. At twenty-one he graduated in letters at Hamilton and five years later in medicine at Fairfield, having attended six courses of lectures. He became at once his father's assistant, and accompanied the latter when he removed his family to Geneva to take the chair of chemistry in the flourishing medical college in that village. In May,

1840, he went to Lockport, and from that time until October, 1841, served as an engineer on the Erie Canal under Alfred Barrett, Chief Engineer on that public work, Western Division. This was a time of great activity and enjoyment. He went up the Genesee Valley Canal as far as Cuba, and made minute examinations of the falls and ravine at Portage. His record book is full of plans, sketches, sections and calculations. In January, 1841, he went to Albany and spent three months in the Canal Office working up reports. Interrupted by an attack of inflammatory rheumatism which recurred again in April—and probably at that remote day the foundation of his ultimately fatal heart disease was laid—he resumed his active work in the Canal Office in May and continued it until he started for the far West, for other and more congenial occupation, for in the fall of 1841 George Hadley was elected Professor of Chemistry in the University of Missouri, situated at Columbia, where he remained for two years. While there he made many sketches with his ready pen and pencil illustrative of the educational surroundings of border life which are now in the possession of his family. They are both instructive and amusing. In 1843-4, after his return from Missouri, Dr. Hadley spent the winter months in New Haven, Conn., attending Silliman's lectures on chemistry and geology and studying geology more particularly. He has left a portfolio of admirable geological drawings made at this time from the works of Agassiz and Forbes on the glaciers, which he studied very thoroughly. It was shortly after this period (1847) that he spent some months in the Lake Superior regions (northern shore), in the service of a Canadian mining company, investigating scientifically the copper and other mineral deposits, and acquiring that precise knowledge of metals and metallurgy for which he was subsequently very frequently consulted. His narratives of his manner of life while there was always extremely interesting, abounding as they did in curious incidents and in vivid scenic descriptions. His tales of his wanderings in birch bark canoes with the Indians as his sole companions, and his enthusiastic reference to the bracing atmosphere and the clear skies and the immense expanse of vision and of the boundless blue waters, sounded like the tales of Cooper or of Scott. It is not strange that in his failing days he went again to this elysium of his youth in hopes of restoration.

In 1846, his father having been appointed Professor of Chemistry in this institution, he was associated with him as lecturer, and two years later succeeded him as Professor, the latter retiring with the well deserved title of Emeritus. This chair he filled, I need not tell how acceptably, during the remainder of his life. Besides discharging his duties here he lectured in Castleton, Vt., from 1856 to 1861, and at Middlebury, Vt., in 1858-59. On January 2, 1855, he was married in New Haven, Conn., to Miss Sophia G. Larned, sister of the eminent Prof. Wm. A. Larned of Yale College—this lady being up to this time teacher of chemistry and other sciences in the celebrated educational school for young ladies kept by Miss Bush of New Haven. From this time forward he spent but little time away from Buffalo. It was his home; and here with his own family and with his aged parents, who lived with him, he delighted to dwell in his modest and comfortable residence in the outskirts of the city. It was near enough for all business purposes, and yet sufficiently removed to allow him to indulge in the pleasure of gardening and horticulture, of which, like his father, he was very fond. Besides discharging all the obligations resulting from his connection with the College he taught his favorite branch while his health permitted in the State Normal School. He became a member of the American Association for the Advancement of Sciences early in its existence, and was among the first to be elected a Fellow of the same. He was also a most valued member of the Buffalo Society of Natural Sciences and President of the Buffalo Microscopic Society. He had no idle moments; he was always busy, and busy about something useful. He published but little, but he wrote and thought much. He has left two thousand closely written pages of notes and observations of most valuable matter which it is hoped may some day be published in whole or in part. There is no waste of words in them, there is a mine of wisdom and of truth. A writer in THE COURIER of Oct. 17th has so well expressed his character that I cannot forbear to quote a portion of his article.

As a lecturer he was in many respects unequaled. Modest and unpretending in manner, he won the confidence and love of those under his instruction; and his clear and convincing demonstrations so appealed to the intelligence of the student and the young physician as to win their very highest regard. He presented nothing as truth that he did not know to be the truth, and by his associate Professors as well as those in whose professional education he was immediately interested he was regarded as infallible in his teachings.

He was distinguished as a medico-legal witness and in this department he was probably without a successful rival in the country. His remarkable faculty for stating the chemical processes by which he reached his conclusions, and the clearness, conciseness and precision of his explanations carried unfailing conviction; and the court records of this and the adjoining counties bear witness to the high regard in which his opinions were held.

An inborn love of truth was the dominating trait in his character, and in his private as in his professional life this declared itself most actively and uncompromisingly. This trait was equalled by his modesty and unobtrusiveness; and his moral and social qualities were on a par with his great intellectual gifts and attainments. He was a model scholar, teacher and Christian gentlemen.

He died on the 16th of October, 1877; and when his demise was announced sadness prevaded this whole city—a light had gone out from it. Every professional man mourned a personal loss. His colleagues convened and took appropriate action, as did also the Medical Society of the county; and the words of commendation of his life, of sorrow for his death and of respect for his memory were no mere conventional formalities, they were earnest and heartfelt. All the day of his funeral the pall of darkness overspread the sky, and the tears of the heavens fell abundantly. It was appropriate; it was from sorrow that so good an example of a man and so wise an expounder of Nature's laws had been taken from this earth. At the obsequies, young gentlemen, many of you performed most appropriate and acceptable service as an honor guard; may the lesson of that hour be stamped upon your hearts forever—and at last, of each one of you may it be said: "Well done, good and faithful servant."

"Can that man be dead, whose spiritual influence is upon his kind?
He lives in glory, and his speaking dust
Has more of life than half its breathing moulds." —*Miss Landon.*

